# Post-mortem interval estimation of human skeletal remains by micro-computed tomography, mid-infrared microscopic imaging and energy dispersive X-ray mapping

**DOI:** 10.1039/c4ay02943g

**Published:** 2015-02-18

**Authors:** S. Longato, C. Wöss, P. Hatzer-Grubwieser, C. Bauer, W. Parson, S. H. Unterberger, V. Kuhn, N. Pemberger, Anton K. Pallua, W. Recheis, R. Lackner, R. Stalder, J. D. Pallua

**Affiliations:** a Institute of Legal Medicine , Medical University of Innsbruck , Müllerstraße 44 , 6020 Innsbruck , Austria . Email: Johannes.Pallua@i-med.ac.at; b Department of Internal Medicine IV (Nephrology and Hypertension) , Medical University of Innsbruck , Anichstraße 35 , 6020 Innsbruck , Austria; c Penn State Eberly College of Science , University Park , PA , USA; d Material-Technology , Leopold-Franzens University Innsbruck , Technikerstraße 13 , 6020 Innsbruck , Austria; e Department of Traumatology , Medical University of Innsbruck , Anichstraße 35 , 6020 Innsbruck , Austria; f Department of Pharmaceutical Technology , Institute of Pharmacy , Leopold Franzens University of Innsbruck , Innrain 52c , 6020 Innsbruck , Austria; g Former Institute for Computed Tomography-Neuro CT , Medical University of Innsbruck , Anichstraße 35 , 6020 Innsbruck , Austria; h Department of Radiology , Medical University of Innsbruck , Anichstraße 35 , 6020 Innsbruck , Austria; i Institute of Mineralogy and Petrography , Leopold-Franzens University Innsbruck , Innrain 52 , 6020 Innsbruck , Austria

## Abstract

In this study different state-of-the-art visualization methods such as micro-computed tomography (micro-CT), mid-infrared (MIR) microscopic imaging and energy dispersive X-ray (EDS) mapping were evaluated to study human skeletal remains for the determination of the post-mortem interval (PMI). PMI specific features were identified and visualized by overlaying molecular imaging data and morphological tissue structures generated by radiological techniques and microscopic images gained from confocal microscopy (Infinite Focus (IFM)). In this way, a more distinct picture concerning processes during the PMI as well as a more realistic approximation of the PMI were achieved. It could be demonstrated that the gained result in combination with multivariate data analysis can be used to predict the Ca/C ratio and bone volume (BV) over total volume (TV) for PMI estimation. Statistical limitation of this study is the small sample size, and future work will be based on more specimens to develop a screening tool for PMI based on the outcome of this multidimensional approach.

## Abstract 2

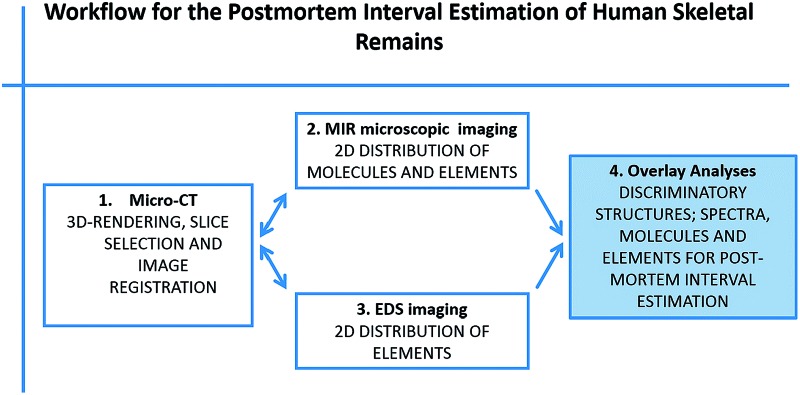
In this study different state-of-the-art visualization methods were evaluated to study human skeletal remains for the determination of the post-mortem interval (PMI).

## 1. Introduction

Post-mortem interval (PMI) estimation of human skeletal remains is a very important, but still unsatisfactorily defined problem. Investigating and legal authorities have major interest in recent skeletal remains, not older than a few decades.^1–3^ The PMI is generally determined from skeletal remains by analyzing morphological appearances, by estimating the time-related deterioration of other elements recovered on the site of discovery (clothing, personal objects, *etc.*), or by using chemical and physical methods.^4^ More likely the time since death estimation is based on the experience of the scientist.^5^ The method involving the assessment of the external changes of bones is probably more reliable than others, even though it is less objective.^6^ The bones themselves can give an idea of the elapsed time since death according to the presence or absence of ligamentous structures and the rates of leaching out of fats and other organic matter.^7^ Many studies focused on the chemical and physical changes of bones after death and on numerous variables influencing the diagenetic process (*e.g.* burial environment, type of bone tissue itself, *etc.*) were performed.^8–10^

Methods of the PMI estimation of human skeletal remains mainly include histological examination,^11–13^ reaction with mineral acids, reaction with benzidine, nitrogen loss,^14^ amino acid content of proteins, serological protein determination,^15,16^ degradation of lipids, remnants of fat-transgression,^17^ UV-fluorescence, radiocarbon methods,^18^ strontium-90 content,^19^ luminol chemiluminescence reaction,^3,20^ X-ray diffraction (XRD),^21^ nuclear magnetic resonance, electron spin resonance, infrared (IR),^22–27^ and Raman spectroscopy.^28,29^ Technical innovations, such as modern radiological procedures,^30^ radioisotopic techniques,^31,32^ IR spectroscopy^27^ and forensic DNA analysis,^33,34^ have now opened up numerous new possibilities in the field of forensic anthropology. Still, there is a need for tools for a rapid and more accurate estimation of the PMI. The usage of state-of-the-art visualization methods for PMI research might provide a useful extension to conventional ways. The primary goals of this study are: (1) investigation of the bone density, (2) analysis of organic and mineral components, (3) determination of elemental composition, and (4) application and comparison of the gained results in combination with partial least squares (PLS) regression for the determination of the PMI. On this account, different imaging techniques are combined for a deeper characterization of human skeletal remains: micro-computed tomography (micro-CT), mid-infrared (MIR) microscopic imaging and energy dispersive X-ray (EDS) mapping followed by multivariate data analyses (MIAs).

## 2 Materials and methods

### 2.1 Sample collection

Randomly chosen paleoanthropological bones from one archeological site and of diverse ages (*n* = 3) and recent forensic bone samples (*n* = 3) were analyzed. The measured human skeletal remains are summarized in Table 1. Typical anthropological methods (*e.g.* pelvic bone, skull characteristics^4,35^) and DNA typing were used for sex determination (data not shown). The part between the upper and mid third of the thigh bone was used for analyses in all cases. From each bone, one transversal section was cut to a thickness of about 7 mm using a vibrating saw. The cutting planes were polished with 1200 grit carbide paper prior to analysis.

**Table 1:** Anthropological properties and the calculated Bone Volume (BV) over Total Volume (TV) of the measured human skeletal remains

Samples	Sex	PMI	BV/TV
Forensic	Male	3 years^*a*^	0.400
Forensic	Male	25 years^*a*^	0.476
Forensic	Male	70 years^*a*^	0.462
Anthropological	Male	650–870 years^*b*^	0.368
Anthropological	Female	1020–1210 years^*b*^	0.350
Anthropological	Male	1030–1260 years^*b*^	0.283

^*a*^Missing person.

^*b*^Radiocarbon dated.

### 2.2 Confocal microscopy – Infinite Focus Microscopic (IFM) imaging data collection

Confocal microscopy tissue imaging measurements were performed on a confocal light microscope type Infinite Focus G4 (Alicona, Graz, Austria) using IFM 3.5.1.5 x 64 software (Alicona, Graz, Austria). Specimens were scanned using a 5× objective, with a vertical resolution of 2 μm and a lateral resolution of 7.8 μm. Images vary in size in order to scan the particular specimen.

### 2.3 Micro-Computed Tomography (micro-CT)

Besides the 3D data acquisition, micro-CT was used to find morphology structures statistically for PMI estimation. Micro-CT experiments were performed with a SCANCO Viva40CT (Scanco Medical AG, Brüttisellen, Switzerland) with an effective 12 μm isotropic voxel size and with 70 kV, 114 μA tube current, 380 ms exposure time, and 2.000 projections. The acquired image stacks have a size of 2048 × 2048 voxels with 16 bit gray value resolution and a stack size dependent on the extension of the object investigated.

#### Imaging data processing and analysis

For data processing and data analysis, the obtained results were transferred to a high-performance 64 bit PC with 32 GB RAM featuring ImageJ (http://rsbweb.nih.gov/ij/), Fiji (; http://fiji.sc/Fiji), and Amira 5.3.3 (FEI Visualization Sciences Group, Mérignac Cedex, France). The software packages were used to perform the measurement routines (ImageJ, Fiji), segmentation (Amira) and volume and density visualization or combination of both (Amira). For analyses of the bone density the BoneJ plugin^36^ and the image processing package Fiji (; http://fiji.sc/) were used. This plugin is designed for a straightforward calculation of the bone volume fraction (BV/TV), which is the volume of the mineralised bone per unit volume of the sample.

### 2.4 MIR microscopic imaging

Mid infrared (MIR) reflection measurements were performed at room temperature using a Bruker Vertex 70 Fourier transform infrared (FTIR) spectrometer, continuously flushed with dried air to minimize the water-vapor background, coupled to a Hyperion 3000 microscope equipped with a usual nitrogen-cooled MCT-D316-025 (mercury cadmium telluride) detector, in the following called single-element detector, and a nitrogen-cooled focal plane array (FPA) detector consisting of 64 × 64 MCT-D364 detectors. Visual image collection was performed *via* a video camera integrated in the microscope stage.

The bone specimens for PMI estimation were imaged with a nominal lateral pixel resolution of 2.65 μm × 2.65 μm using the FPA detector, as well as 70 μm × 70 μm and 140 μm × 140 μm per pixel for each spot by mapping using the single element detector. MIR (detector range: a) detection for FPA measurements was carried out in the spectral range of 4000 cm^–1^ to 750 cm^–1^, and single element mapping measurements were recorded from 4000 cm^–1^ to 550 cm^–1^. Spectra were recorded with a spectral resolution of 4 cm^–1^ with 20 co-added scans. Before each sample measurement, a background spectrum of a gold-coated substrate was recorded with a spectral resolution of 4 cm^–1^ with 30 co-added scans. The scan number and spectral resolution were optimized to get a good signal to noise ratio of the recorded spectra. For detailed information about detector theory, technology and current developments the interested reader is referred to the cited literature.^37–39^ After measurements, the specimen was scanned with an IFM for histological reevaluation and comparison to the imaging results by a forensic doctor.

#### Data processing

All spectral data processing and image assembly were performed using OPUS 6.5 software (Bruker), The Unscrambler X 10.2 (Camo, Norway, Oslo) and CytoSpec™ software package (http://www.cytospec.com, Hamburg, Germany). Univariate chemical maps depicting a single spectral feature and multivariate imaging analysis (MIAs) were generated by using CytoSpec™ software.

#### Principle component analyses (PCA)

Atmospheric absorptions and noise were removed using the OPUS 6.5 (Bruker) software prior to principle component analyses (PCA) and image analysis. After atmospheric correction and noise reduction, PCA models were generated using The Unscrambler X 10.2 software. For PCA model generation, tissue type-associated spectra were selected using the CytoSpec™ software. For this purpose, a forensic doctor evaluated the sample and defined the regions of interest (ROIs). The extracted spectra of ROIs were imported into The Unscrambler X 10.2 software and underwent several data pretreatments (*e.g.*, baseline correction, normalization), before PCA model generation.

#### MIR image analysis

Initially, atmospheric correction and noise reduction of the MIR microscopic imaging and mapping data were performed by using the OPUS 6.5 software (Bruker). After this spectral treatment, the datasets were loaded in the CytoSpec™ software. Spectra were pre-processed by applying second derivative (Savitzky–Golay algorithm, 13 smoothing points) and vector normalization in the wavenumber range of 4000 cm^–1^ to 750 cm^–1^ for FPA results and 4000 cm^–1^ to 550 cm^–1^ for mapping results. These procedures led to higher resolved peaks, eliminated background slopes and reduced the influence of intensity changes caused by differences in tissue density and tissue roughness.^40^ The processed spectral datasets were used for subsequent univariate image analyses. Furthermore, the imaging results of univariate image analyses were assembled and compared directly with the IFM images captured from the same samples.

### 2.5 Energy Dispersive X-ray (EDS) mapping

EDS measurements were performed on a scanning electron microscope (SEM) type Quanta 3D 200 DualBeam (FEI, Eindhoven, Netherlands) using xTm 1.72 software. An EDS-detector type Saphire Si(Li) (EDAX) is coupled, which has an area of 10 mm^2^ and a Super Ultra Thin window (SUTW) to analyse light elements down to beryllium. The detector was controlled by the software Genesis Spectrum V.6.29. For SEM data acquisition a large field detector (LFD) and a pressure of 0.3 mbar were chosen. Images were taken at a magnification of 150×, a half field with of 2.03 mm and an acceleration voltage of 15 kV. For EDS mapping a resolution of 1024 × 1024 pixel with a dwell time of 200 μs was selected. Each measurement was build-up of 4 frames. For every specimen three equally sized areas were scanned. To calculate ratios between different elements, each sample was measured six times. Therefore, the same SEM conditions were used. The EDS measurement time was 23 seconds.

### 2.6 Multivariate data analysis

To evaluate the ability of MIR microscopic measurements to predict the bone volume (BV) over total volume (TV), the calcium to phosphorus (Ca/P) ratio, the calcium to carbon (Ca/C) ratio and the sum of Ca and P (Ca + P) for the determination of the PMI, partial least-squares (PLS) regression models were developed. Data analyses were performed using The Unscrambler X 10.2 (Camo, Oslo, Norway) software. MIR microscopic imaging spectra were log 1/*R* transformed in the first step and an average spectrum for each sample was recorded. Different pre-treatments like normalization, 1st to 2nd derivative (Savitzky Golay algorithm),^41^ and standard normal variate (SNV)^42^ were systematically applied on the spectra to reduce systematic variations. Wavenumber regions with higher noise or low information content were removed and leave-one-out full-cross-validation (CV) was carried out for the MIR spectra. The accuracy and quality of the PLS models were evaluated by using the root mean square error of cross validation (RMSECV), the standard error of cross validation (SECV), *R* ^2^, the number of factors, and the ratio performance deviation (RPD). The RPD is the ratio of the standard deviation of the sample-values to the SECV and can therefore be seen as a quality factor for the interpretation model. Values higher than 2.5 are acceptable, values >5 are adequate for quality control, and RPDs > 10 are considered to be excellent.^43^

## 3 Results and discussion

The main focus of this first study was the optimization of the parameters that allow the estimation of the PMI of human bones using different imaging techniques, namely radiological, molecular imaging and bioinformatics technologies. The integration of structural images (obtained by micro-CT and IFM), semi-quantitative distribution/location of defined substance classes (generated by MIR microscopic measurements) as well as element distribution patterns (generated by EDS mapping) represent a powerful platform for the characterization of human skeletal remains.

**Fig. 1:**
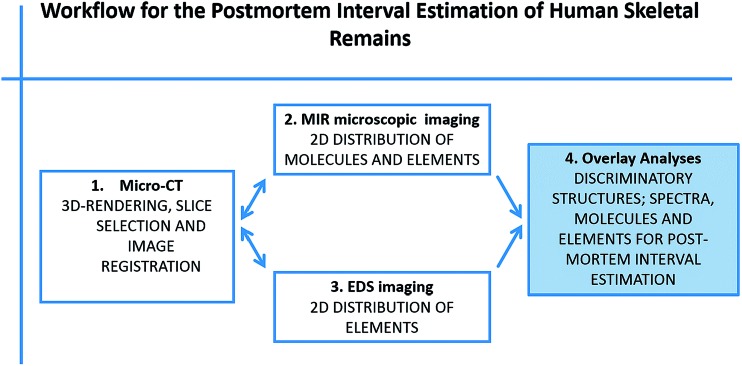
Overview of the workflow for the PMI estimation of human skeletal remains. (1) Micro-CT measurements. (2) MIR microscopic and (3) EDS measurements and data processing. (4) Multivariate data analyses.

In total 6 human bone samples were investigated according to the work flow presented in Fig. 1.

### 3.1 Morphological analysis *via* micro-CT

**Fig. 2:**
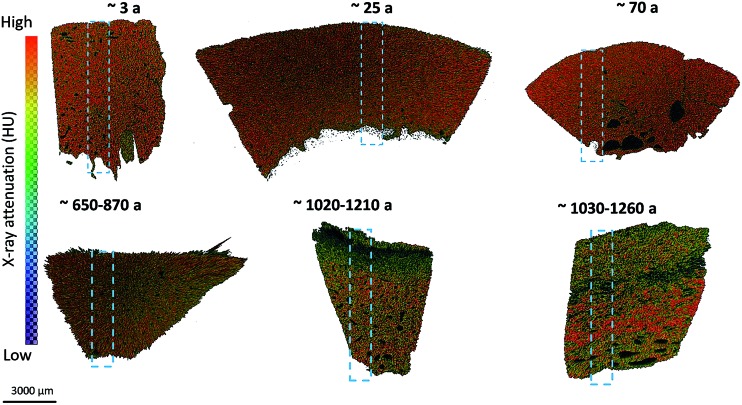
Color-coded micro-CT images with ROIs for bone density measurement, MIR microscopic imaging, and EDS mapping. Phantom-based calibration to Hounsfield unit (HU).

Micro-CT was used to demonstrate the influence of PMI on the bone density and to evaluate how far MIR reflection spectra enable a match of the micro-CT results. Analyses of the resulting micro-CT datasets were performed using the aforementioned software packages. The results are illustrated in Fig. 2 and Table 1. Due to the fact that MIR microscopic reflection imaging is limited to surfaces and micro-CT provides images of the internal structure, the best fitting micro-CT image slice was selected manually including an oblique reformatting of the image stack being as close as possible to the surface and preserving an appropriate image quality at the same time.

Two specimens of the paleoanthropological bones (all radiocarbon dated) with an almost equal PMI indicated an equivalent bone density. In contrast, measurements on specimens of younger age displayed a higher value of bone density. According to our hypotheses, the most recent three specimens (3–70 a PMI) revealed a much higher bone density (Fig. 2). The results are in accordance with the hypotheses from O. Gaber, published in D. Leopold.^44^

### 3.2 MIR microscopic imaging

**Fig. 3:**
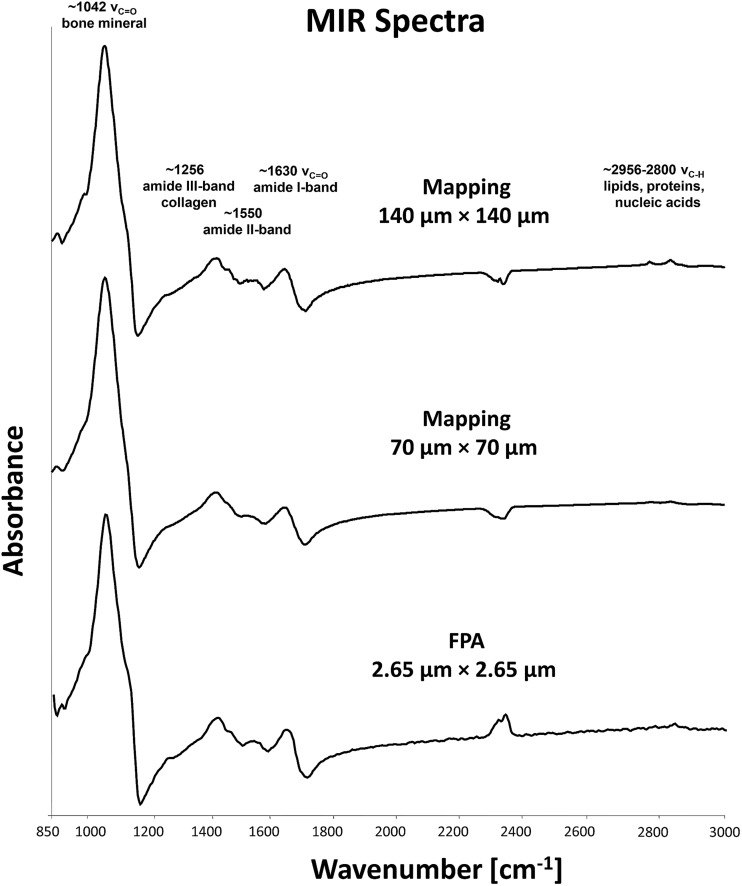
IR spectra from a bone sample, displayed in the range of 4000 cm^–1^ to 850 cm^–1^. Major absorption bands are indicated. Spectra demonstrate a slight increase in noise as the aperture size decreases. The C–H stretching vibrations between 2956 cm^–1^ and 2800 cm^–1^ can only be recorded by mapping with an aperture size of 140 μm × 140 μm. The absorption feature between 2300 and 2400 cm^–1^ is due to atmospheric CO_2_ and does not interfere with bands relevant in this study.

To illustrate the effects of the beam focus on spectra quality, representative spectra chosen from the same region of one individual bone sample recorded by the two methods are compared (Fig. 3). It can be demonstrated, that there is no detrimental effect of the measurement settings on the spectra quality in the range of 1800 cm^–1^ to 850 cm^–1^. The C–H stretching vibrations of phospholipids, protein side chains as well as nucleic acid sugars between 2956 cm^–1^ and 2800 cm^–1^ can only be recorded by mapping with an aperture size of 140 μm × 140 μm. Therefore, further mapping measurements were performed with an aperture size of 140 μm × 140 μm. With this measurement mode it was possible (a) to record the C–H stretching vibrations between 2956 cm^–1^ and 2800 cm^–1^, (b) to reduce the scan time, (c) to collect spectra in the detector range of 4000 cm^–1^ to 550 cm^–1^ and (d) to measure large area of the sample, which simplified a correlation with micro-CT results.

**Fig. 4:**
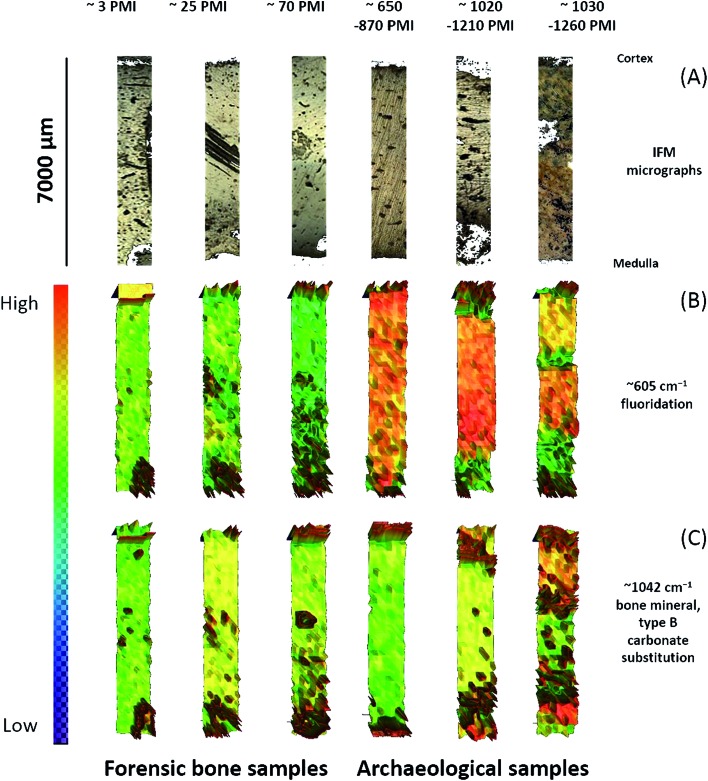
(A) IFM micrographs of individual patient's tissue samples. (B and C) Infrared spectroscopic maps of the bone surface obtained for the fluoridation at 605 cm^–1^ and bone mineral at 1042 cm^–1^.

**Fig. 5:**
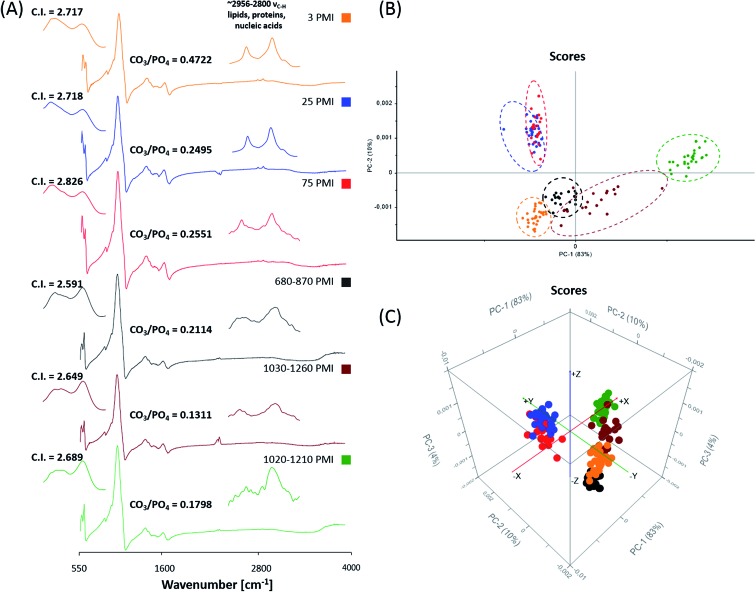
(A) Representative MIR spectra of forensic and archaeological bone samples with the corresponding C.I. and CO_3_/PO_4_ value. 2-D and 3-D Score plots of MIR spectra in the region of 1700 cm^–1^ to 550 cm^–1^ (B and C). For the differentiation between forensic and archaeological bone samples for each bone sample 30 spectra were selected from degradation free regions. Each data point represents one spectrum of the respective (colour coded) bone sample.

Analyses of the resulting MIR microscopic imaging datasets were performed using the mentioned software packages. In this study, 6 bone samples with different PMIs were analysed by individual chemi-map representations, spectra-analysis and principle component analyses (PCA) (Fig. 4 and 5).

The results from six individual bone samples by chemi-maps are illustrated in Fig. 4. The output of the data analyses illustrates the ability of spectroscopic imaging to reflect decomposition/aging processes of bone samples with a nominal lateral resolution of 140 μm × 140 μm per pixel for each spot. The images in Fig. 4(A) represent micrographs of the bone samples, which were measured afterwards by MIR microscopic imaging. These micrographs serve as comparison with images constructed from chemical maps (Fig. 4(B) and (C)). Fig. 4(B) depicts chemical maps generated by integrating the area under the band absorption at 605 cm^–1^, which is an indicator of fluoridation. The results demonstrate that archaeological bones exhibit higher fluoridation than forensic ones. The chemical maps of the absorption at 1042 cm^–1^ is attributed to bone minerals (Fig. 4(C)), indicating an inhomogeneous distribution. From the results, it may be assumed that the peak at 1042 cm^–1^ is not suitable for PMI estimation compared to the peak at 605 cm^–1^.

Fig. 5(A) displays MIR (detector range set from 4000 cm^–1^ to 550 cm^–1^) reflection spectra of the 6 bone samples. Prominent features in spectra of archaeological samples are absorption bands at 605 cm^–1^ (indicator of fluoridation) and at 1042 cm^–1^ (indicator of bone mineralization). Forensic bone samples exhibit more organic bands in contrast to archaeological ones, which could be demonstrated by the C–H stretching vibration between 2956 cm^–1^ and 2800 cm^–1^. Additionally, the crystallinity index (C.I.) and the CO_3_/PO_4_ index were calculated from baseline-corrected spectra as suggested.^22,27,45–48^ The C.I. and CO_3_/PO_4_ values of forensic skeletal remains are 2.59–2.84 (average 2.71 ± 0.12) and 0.25–0.47 (average 0.32 ± 0.12) and for archaeological samples are 2.59–2.69 (average 2.64 ± 0.05) and 0.18–0.21 (average 0.17 ± 0.04). The results are consistent with the findings of Patonai *et al.* ^27^ During the age-related degradation, the C.I. index increases with the time as the apatite undergoes a change, to larger and more ordered crystals, as well as a change in the carbonate to phosphate ratio could be observed.

For further spectra analysis, principal component analyses were performed to fully characterize the range of spectral variations. With PCA the dimensionality of MIR microscopic imaging spectra is reduced while as much information as possible is retained. The scores of the first principle components are used to generate meaningful plots without a detailed understanding of the underlying sample biochemistry. For the PCA across 6 different bone samples, 30 spectra were chosen. The results of spectral analyses using PCA are illustrated in Fig. 5(B) and (C).

The score plot of the first and the second principal component is based on 30 spectra of the specimen. For deploying PCA models, reflection spectra were transformed to log(1/*R*). Additional pre-treatments for MIR spectra such as baseline offset and area normalization were utilized. The score plot in Fig. 5(B) shows that a 2-D and 3-D visualization of spectral clusters for principal components 1 and 2 explains 93% of the total variance and can separate the different bone samples. PCA models indicate that most of the descriptive information can be found in the region from 1700 cm^–1^ to 550 cm^–1^. This statistical strategy allows an easy feature extraction of several datasets. However, it could not be determined to what extent this variation is caused by qualitative and/or quantitative alterations.

### 3.3 Energy Dispersive X-ray (EDS) mapping

**Fig. 6:**
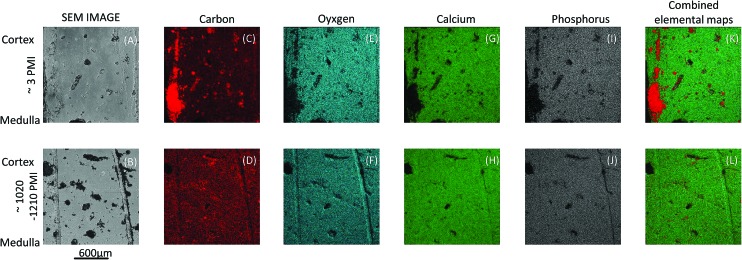
SEM secondary electron (SE) images and EDS data of one forensic (A, C, E, G, I and K) and one archaeological bone sample (B, D, F, H, J and L). EDS maps of elemental distribution: carbon (C and D) in red, oxygen (E and F) in turquoise, carbon (G and H) in green and phosphate (I and J) in grey. Combined elemental maps (K and L) presented as false colours: carbon, oxygen, carbon and phosphorus.

Energy dispersive X-ray (EDS) mapping was used to decipher the elemental distribution pattern and to determine the calcium to phosphorus (Ca/P) ratio as well as the calcium to carbon (Ca/C) ratio of the bone sample by the sum of Ca and P (Ca + P) on the six bone samples. Exemplarily, mapping results from two individual bone samples are illustrated in Fig. 6. The output of the data analyses illustrates the ability of EDS mapping to reflect decomposition/aging processes of bone samples and the elemental distribution within the bone specimen. The images in Fig. 6(A) and (B) represent SEM secondary electron (SE) images of the bone samples, which were simultaneously measured during EDS mapping. Micrographs serve as reference to elemental maps (Fig. 6(C)–(J)) and combined elemental maps (Fig. 6(K and L)). Fig. 6(C) and (D) depict elemental maps of carbon, which is an indicator of organic matter. The results demonstrate that forensic bones exhibit regions with a higher content of carbon than archaeological ones. Fig. 6(K) and (L) depict combined elemental maps. The displayed images represent a combination of carbon (red pixels), oxygen (turquoise pixels), calcium (green pixels) and phosphorus (grey pixels). The principal correspondence between the SE images and the combined elemental maps is obvious and it is possible to correlate elemental signals detected by the used method with decomposition/aging processes.

**Fig. 7:**
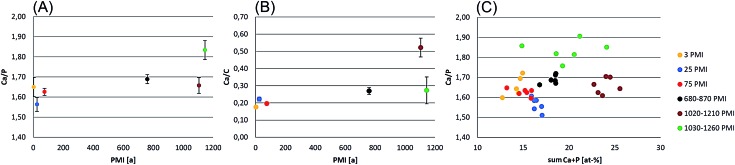
Calculation of calcium to phosphorus (Ca/P), calcium to carbon (Ca/C) ratio and the sum of calcium and phosphorus (Ca + P) for the determination of bone decomposition/aging. (A) Calcium to phosphorus (Ca/P) ratio: Ca/P increases with PMI. (B) Calcium to carbon ratio increases with PMI. (C) The C + P was used to determine the mineralogical content, which increases with PMI.

For each bone the atomic-composition of six areas was determined. These results were used to calculate the calcium to phosphorus (Ca/P) ratio, the calcium to carbon (Ca/C) ratio and the sum of Ca and P (Ca + P) for the determination of bone decomposition/aging. The results in Fig. 7 demonstrate the Ca/P ratio, Ca/C ratio and Ca + P. It could be demonstrated that the Ca/P and the Ca/C increase per PMI. The Ca/P ratio of the forensic bone sample is consistent with a theoretical molar ratio of 1.67 already reported in the literature.^49,50^ During degradation a tendency to higher Ca/P ratios can be observed. To support our theory it is advised to investigate more samples. More significant is the increase of Ca/C, which demonstrates the degradation of organic matter. This degradation results in a higher mineralogical content, illustrated by the sum of calcium and phosphorus (Fig. 7) and exhibits a higher content with increasing PMI.

### 3.4 Multivariate data analysis of the obtained data

**Fig. 8:**
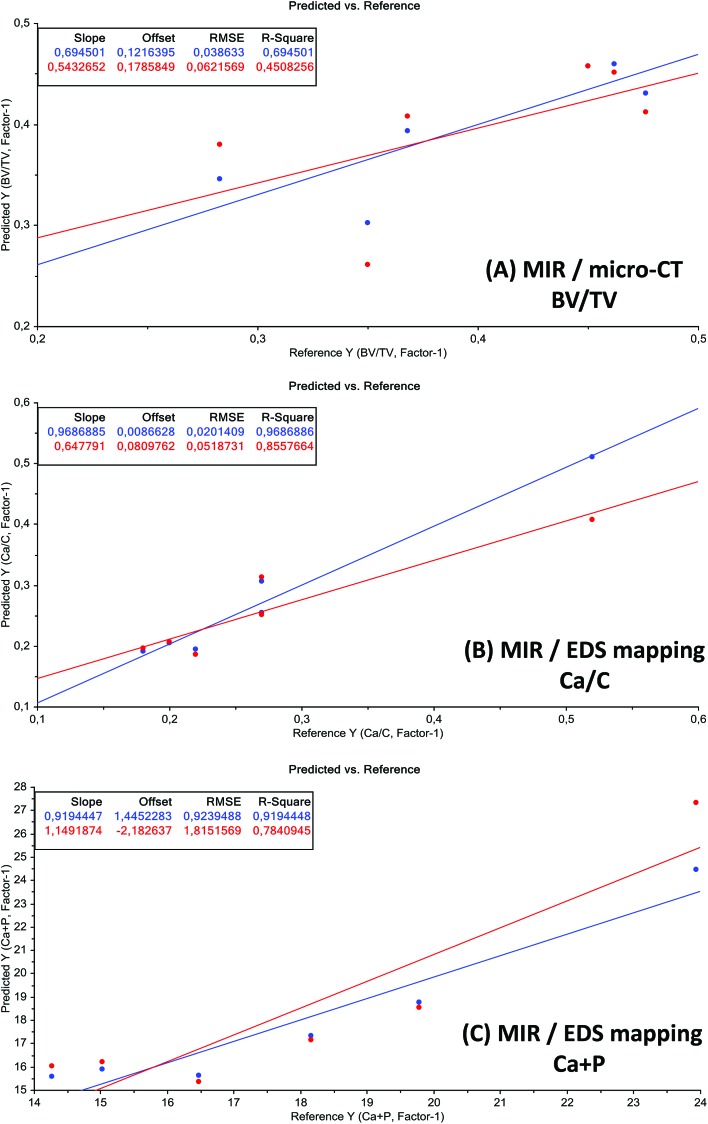
Predicted *vs.* reference plots of the best test-set validation models. Unit of *x*- and *y*-axis is %. Blue: calibration and red: validation.

To evaluate the ability of MIR microscopic measurements to predict the bone volume (BV) over total volume (TV), the calcium to phosphorus (Ca/P) ratio, the calcium to carbon (Ca/C) ratio and the sum of Ca and P (Ca + P) for the determination of the PMI, partial least-squares (PLS) regression models were developed. For this purpose different data pre-processing algorithms such as normalization, standard normal variate (SNV), and 1st to 2nd derivatives were applied on average MIR spectra to reduce the influence of systematic disturbances. The best calibration for each model was achieved with normalized (NORM) spectra. Quality parameters of the best PLS models are listed in Table 2. Wavenumber regions taken for PLS model calculations were 1155 cm^–1^ to 622 cm^–1^ and 2970 cm^–1^ to 2830 cm^–1^. Weighted regression coefficients of the PLS models indicate that wavenumbers from 1155 cm^–1^ to 622 cm^–1^ have the highest influence on the PLS model for PMI estimation. The predicted *versus* reference plots of the best models are depicted in Fig. 8. The MIR microscopic imaging presented an acceptable calibration quality for the prediction of the calcium to carbon (Ca/C) ratio and bone volume (BV) over total volume (TV). The sum of Ca and P (Ca + P) validation revealed a RPD of 0.36, which is clearly below the stated value of 2.5. Furthermore, it could not clearly be evaluated, which is the best pre-treatment for predicting the calcium to phosphorus (Ca/P) ratio.

**Table 2:** PLS regression results for MIR microscopic imaging: root mean square error of cross validation (RMSECV), standard error of cross validation (SECV), *R* ^2^ and calculated ratio performance deviation (RPD). Best calibrations are highlighted in bold. *R* ^2^ refers to validation

	RMSECV	SECV	*R* ^2^	RPD
**MIR/micro-CT BV/TV**	0.0621	0.0679	0.4508	6.64
**MIR/EDS mapping Ca/C**	0.0518	**0.0539**	**0.8557**	15.8
**MIR/EDS mapping Ca + P**	1.8151	2.1981	0.7840	0.36

## 4. Conclusions

In this first evaluation different state-of-the-art visualization methods such as micro-computed tomography (micro-CT), mid-infrared (MIR) microscopic imaging and energy dispersive X-ray (EDS) mapping were used to analyse, (1) bone density, (2) organic and mineral components, (3) elemental composition, and finally (4) to compare the gained results in combination with partial least squares (PLS) regression for the determination of the post-mortem interval (PMI) of human skeletal remains. Coupling of micro-CT, MIR microscopic imaging and energy dispersive X-ray (EDS) with multivariate data analysis greatly extends the solitary capabilities of each technique for a deeper characterization of bone samples.

Micro-CT results point out that specimens with a short PMI reveal a higher value of bone density than specimens with a long PMI. This is in accordance with the hypotheses and with the already published literature.^44,51–53^

It could be demonstrated that MIR imaging is a powerful tool, which can be used to assemble chemical maps from bone surfaces. The results illustrate the possibility of obtaining reproducible high quality IR-spectra from bone samples and to display the local distribution of organic and mineral components. Many features of the bone surface that can be visualized microscopically can be displayed in the chemical map representation. The MIR imaging results clarify that organic and mineral components could be imaged semiquantitatively. Thus, MIR imaging, as an analytical and non-destructive technique, could provide information about the molecular structure of the sample prior to destructive DNA extraction. Moreover, the results point out that a short PMI reveal a higher C.I. and CO_3_/PO_4_ values than specimens with a long PMI, which is in accordance with the already published literature.^27^ For further spectral analysis, PCA were performed to fully characterize the range of spectral variations, indicating that most of the descriptive information can be found in the region from 1700 cm^–1^ to 550 cm^–1^. This statistical strategy allows an easy feature extraction of several datasets and fast estimation of the PMI.

The EDS results demonstrate that Ca/P increases with the progression of PMI. In addition, it could be demonstrated that Ca/C increases during the degradation of organic matter, resulting in a higher mineralogical content.

The combination of the used methods enables us to gain a more distinct picture concerning processes during the PMI as well as a more realistic approximation of the PMI based on MIAs. Calculations of the MIR microscopic imaging data presented an acceptable calibration quality for the prediction of the calcium to carbon (Ca/C) ratio and bone volume (BV) over total volume (TV). The prediction of Ca and P (Ca + P) could not be achieved with a reliable accuracy. Statistical limitation of this study is the small sample size, and future work will be based on more specimens to develop a screening tool for PMI based on the outcome of this multidimensional approach.

## Abbreviations

CCD: Charge coupled device
CT: Computed tomography
EDS: Energy dispersive X-ray
FTIR: Fourier transform infrared
FCM: Fuzzy C-means
FPA: Focal plane array
HCA: Hierarchical cluster analysis
HU: Hounsfield unit
IFM: Infinite focus microscopy
IR: Infrared
KMC: K-means clustering
LFD: Large field detector
micro-CT: Micro-computed tomography
MIR: Mid infrared
MIAs: Multivariate imaging analysis
MCT: Mercury cadmium telluride
PCA: Principle component analyses
PMI: Post-mortem interval
PLSR: Partial least squares regression
ROIs: Regions of interest
SEM: Scanning electron microscope
